# Synchronous Multicentric Giant Cell Tumour of Distal Radius and Sacrum with Pulmonary Metastases

**DOI:** 10.1155/2015/354158

**Published:** 2015-05-27

**Authors:** Varun Sharma Tandra, Krishna Mohan Reddy Kotha, Moorthy Gadisetti Venkata Satyanarayana, Kali Varaprasad Vadlamani, Vyjayanthi Yerravalli

**Affiliations:** Department of Orthopaedics and Traumatology, Osmania Medical College, Osmania General Hospital, Afzalgunj, Hyderabad, Andhra Pradesh 500012, India

## Abstract

Giant cell tumour (GCT) is an uncommon primary bone tumour, and its multicentric presentation is exceedingly rare. We report a case of a 45-year-old female who presented to us with GCT of left distal radius. On the skeletal survey, osteolytic lesion was noted in her right sacral ala. Biopsy confirmed both lesions as GCT. Pulmonary metastasis was also present. Resection-reconstruction arthroplasty for distal radius and thorough curettage and bone grafting of the sacral lesion were done. Multicentric GCT involving distal radius and sacrum with primary sacral involvement is not reported so far to our knowledge.

## 1. Introduction

Giant cell tumour (GCT) of bone accounts for about 5% of all primary bone tumours in adults. It occurs most commonly in third to fifth decades of life and affects both males and females with slight female preponderance [1, 2]. Giant cell tumours usually occur as a solitary lytic lesion in the ends of long bones, often extending to subarticular location. Multicentric presentation accounts for less than 1% of GCTs of bone [2]. Sacrum is the commonest site in the axial skeleton for solitary GCTs but its involvement in multicentric GCT (MCGCT) is extremely rare [3, 4]. GCTs are generally considered as benign tumours; less than 2% of cases have pulmonary metastases, which are benign in nature [2].

## 2. Case Report

**Figure 1:**
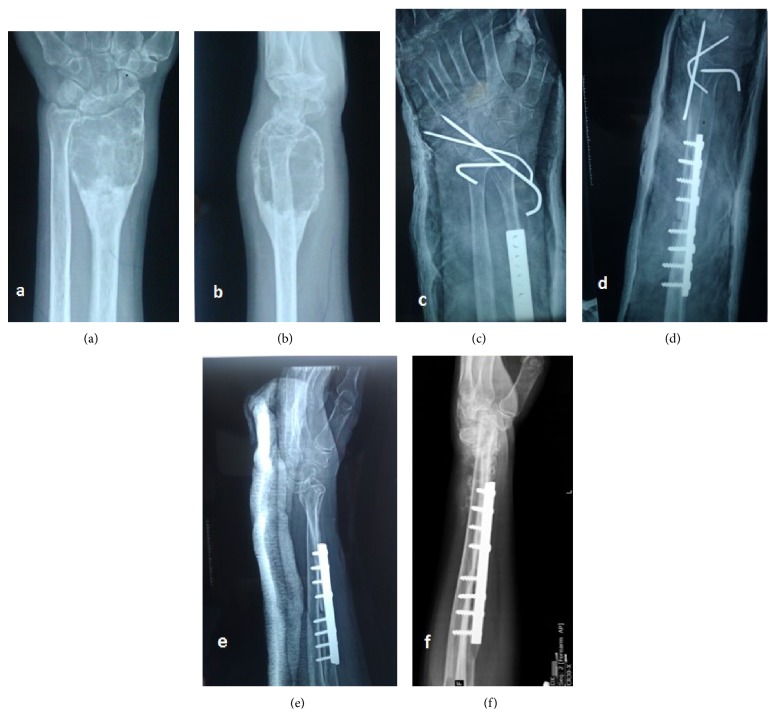
(a and b) Preop plain radiograph of left wrist showing expansile solitary lytic lesion involving left distal radius; (c and d) postoperative radiographs after resection and reconstruction arthroplasty of left wrist; (e) radiographs of her left wrist showing wrist subluxation following k-wire removal; (f) 1 year follow-up radiograph of her left wrist with gross subluxation of wrist.

We report a case of a 45-year-old female who presented to us with a swelling in her left wrist of about one-year duration. The plain radiograph showed a subarticular expansile lytic lesion with “*soap-bubble*” appearance involving distal radius epiphysis with metaphyseal extension and with well-defined margins, suggestive of GCT (Figures 1(a) and 1(b)). Further on the skeletal survey an osteolytic lesion was noted in her right sacral ala. Biopsy of both lesions was performed, and it revealed both lesions as GCT (Figure 4(b)). Only lesion in her wrist was operated on initially, and the sacral lesion was left untreated as the patient was unwilling to undergo major surgery on her back.

“Resection-reconstruction arthroplasty” of distal radius was done using autogenous nonvascularized proximal fibula (Figures 1(c) and 1(d)). The graft was fixed to radius proximally with narrow dynamic compression plate and distally to the ulna with K-wires. In the follow-up, K-wires were removed and the patient was doing well for six months after which she was lost to follow-up.

Twelve months later, she came back to us with complaints of low back pain for three months. Pain was disabling at the time of presentation. There was no history of motor weakness or paraesthesias in her lower limbs, bladder bowel disturbances, or any fever. She could sit and walk with the help of support with difficulty.

**Figure 2:**
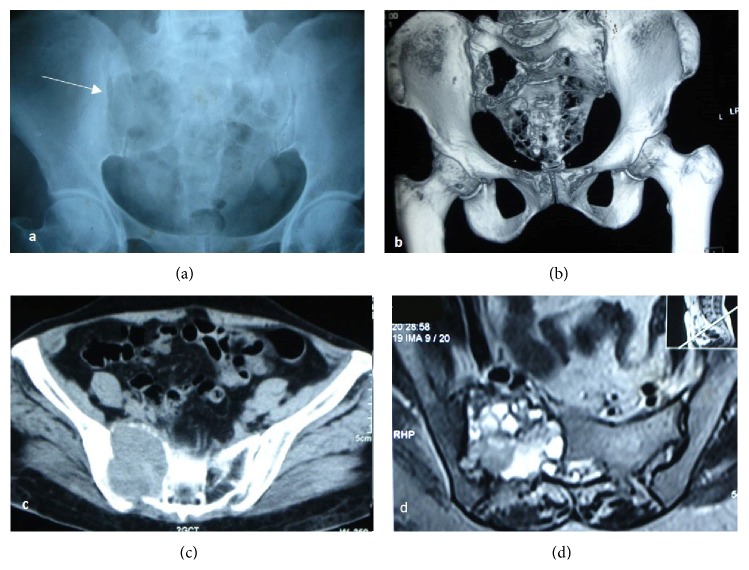
(a) Radiograph of the pelvis AP view; (b) 3D reconstruction and (c) axial section of CT showing expansile lytic lesion involving the right sacral ala with cortical break and surrounding soft-tissue component extending into right SI joint space and pelvis measuring 5.2 × 5.7 × 6.3 cm eroding the adjacent iliac bone. Mass extending into the sacral canal. (d) MRI showing large expansile heterogeneous lobulated lesion with few septations involving right sacroiliac joint. Predominantly lytic lesion with cortical destruction features suggestive of GCT.

On physical examination, no swelling or local signs of inflammation were noted in her low back. Tenderness was noted in the sacrum and right sacroiliac joint. We ordered for a radiograph of pelvis in which we found a large expansile lytic lesion involving body of sacrum and right sacral ala and breach of sacroiliac joint was noted (Figure 2(a)). Further CT with three-dimensional reconstruction was done to delineate the lesion (Figure 2(b)).

**Figure 3:**
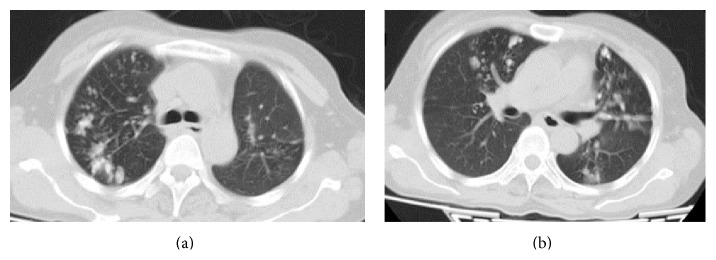
Multiple irregular opacities in apical segments of right upper lobe and left lingular superior segment, anterobasal and posterobasal segments suggestive of metastasis.

The review radiographs of her left forearm showed wrist subluxation (Figures 1(e) and 1(f)), though the patient had no complaints in her wrist. Skeletal survey and screening radiographs showed suspicious lesions in her lung fields which turned out to be pulmonary metastases on Contrast Enhanced CT (CECT) of chest (Figure 3). CECT of brain and abdomen were normal. Technetium^99^ whole body bone scan showed hotspots in the sacrum and both lungs; there was no evidence of recurrence at distal radius.

**Figure 4:**
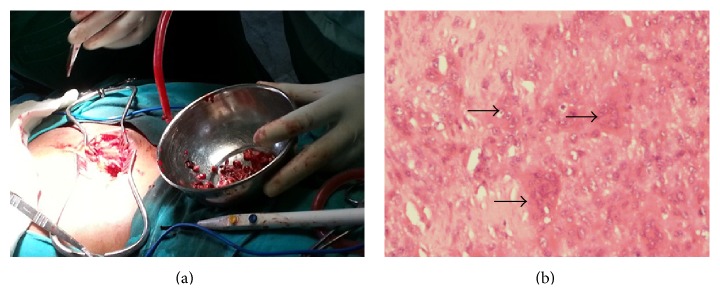
(a) Intraoperative photograph of curettage and bone grafting of the sacral lesion; (b) histopathology slide showing characteristic osteoclast like giant cells (arrows) in the background of mononuclear cells.

After optimizing the patient, sacral lesion was treated by thorough intralesional curettage and bone grafting (Figure 4(a)). The procedure relieved patient of severe episodic pain. The patient developed superficial surgical site infection which later went on to heal with regular dressings and antibiotics. Patient is clinically asymptomatic at one-year follow-up. Pulmonary metastases could not be evaluated further or treated as the patient was unwilling for any intervention.

## 3. Discussion

Less than 1% of cases of GCT are multicentric in origin [1, 4–10]. Most of the cases of MCGCT are reported as single case or as small series [2, 5–15]. Hoch et al. published the largest known series of 30 cases of MCGCT in 2006 [2]. Dhillon and Prasad [4] in 2007 published a comprehensive review on “Multicentric giant-cell tumor of bone” where a total of 101 cases reported so far worldwide were described.

Hoch et al. classified tumours as being “synchronous” when multiple tumours had been discovered at the initial presentation or when a second tumour had been diagnosed within six months after the first. If the second tumour developed more than six months after the first lesion, the lesions were considered to be “metachronous” [1, 2, 16]. In our patient, sacral lesion was found incidentally on the skeletal survey at the initial presentation and is hence classified as synchronous.

Most of the case series reported occurrence of MCGCT at a younger age when compared to solitary GCT [8, 14, 16]. The mean age of multicentric GCT was found to be 22.5 years [4]. Distal radius and sacrum are among the common sites for solitary GCT in the appendicular and axial skeletons, respectively. The age of our patient and the sites of tumor are typical of solitary giant cell tumour, which typically presents in third to fifth decades of life [2].

Though the commonest site of occurrence for the MCGCT is long bones around the knee [2, 4], it was found that they tend to develop more frequently in atypical sites like the small bones of hands and feet and at atypical locations like metaphyseal and metadiaphyseal regions in the bone [2, 4]. Though sacrum happens to be the most common site in the axial skeleton for solitary GCT [3], curiously, the bones of pelvis and spine above the sacrum were reported to be involved frequently in MCGCT, and sacral involvement was found to be extremely rare.

To our knowledge, only a few cases are reported so far worldwide where the sacrum was involved in a case of MCGCT [6, 11, 15]. In none of the cases reported, sacral involvement was noted in the first presentation. Sacral GCT is asymptomatic initially, slow growing, and often diagnosed late when it attains a huge size [17]. Sacral GCT though was incidental finding at initial presentation.

The differences in predilection for age and site for multicentric GCT from solitary GCT suggest that probably a different pathogenetic mechanism of origin is involved in multicentric GCT when compared to solitary GCT. The possible mechanisms of multicentricity suggested include direct extension to contiguous bone, metastasis, and multiple independent foci of disease; the last of them might be true in our case.

The histological appearance of lesions of multicentric GCT is similar to that of solitary GCT of bone [2, 5]. The differential diagnosis for such polyostotic lesions includes brown tumours (hyperparathyroidism), multifocal osteomyelitis (cystic type of tuberculosis), metastasis, multiple myeloma, and some other primary osseous tumours with a giant cell component, such as fibrosarcoma, osteosarcoma, angiosarcoma, and chondroblastoma. The possibility of preexisting systemic conditions in which GCT of bone is known to arrive, such as Paget's disease, must also be considered. Biopsy is considered essential for the diagnosis of GCT.

GCT of the sacrum has higher local recurrence rate than in any other skeletal location; it is found to be as high as 33.9% [17].* En bloc* resection, though effective at preventing recurrence, is associated with greater morbidity to patient [17]. Intralesional curettage followed by intracavitary inactivation of the lesion is chosen rather than* en bloc* resection, which has several complications. Medical management with denosumab or bisphosphonates were not considered as they are largely experimental, though evidence in their favour is building up their optimal dosing schedule and duration of treatment is yet to be established.

Tumour recurrence is a common problem with GCT when compared to pulmonary metastasis [1, 2, 5]. Recurrence is dependent on the type of treatment given to GCT [2]. Pulmonary metastasis is reported only rarely in case of multicentric GCT [2]. Pulmonary metastasis was not found in the case series of sacral GCT reported by Balke et al. [3].

## 4. Conclusion

In summary, GCT of multicentric origin is uncommon and sacral involvement in a case of MCGCT is extremely rare and is reported only a few times in the literature. Ours is the first case of MCGCT where sacral involvement is noted in the first presentation. MCGCTs are known to occur in young females, at atypical sites in the body and at atypical locations in the bone. Presentation in the age group and location typical for solitary GCT cannot rule out chance of another synchronous or metachronous focus of GCT.
